# Mitochondria-mediated ferroptosis contributes to the inflammatory responses of bovine viral diarrhea virus (BVDV) *in vitro*

**DOI:** 10.1128/jvi.01880-23

**Published:** 2024-01-16

**Authors:** Zhijun Li, Bao Zhao, Ying Zhang, Wenqi Fan, Qinghong Xue, Xiwen Chen, Jingyu Wang, Xuefeng Qi

**Affiliations:** 1 College of Veterinary Medicine, Northwest A&F University, Yangling, Shaanxi, China; 2 Key Laboratory of Ruminant Disease Prevention and Control (West), Ministry of Agriculture and Rural Affairs, Xi'an, China; 3 Shaanxi Animal Disease Control Center, Xi'an, China; 4 China Institute of Veterinary Drug Control, Beijing, China; 5 Animal Disease Prevention and Control, Healthy Breeding Engineering Technology Research Center, Mianyang Normal University, Mianyang, Sichuan, China; University of Michigan Medical School, Ann Arbor, Michigan, USA

**Keywords:** BVDV, ferroptosis, mitochondria damage, mitophagy, inflammatory responses

## Abstract

Bovine viral diarrhea virus (BVDV) belongs to the family Flaviviridae and includes two biotypes in cell culture: cytopathic (CP) or non-cytopathic (NCP) effects. Ferroptosis is a non-apoptotic form of programmed cell death that contributes to inflammatory diseases. However, whether BVDV induces ferroptosis and the role of ferroptosis in viral infection remain unclear. Here, we provide evidence that both CP and NCP BVDV can induce ferroptosis in Madin-Darby bovine kidney cells at similar rate. Mechanistically, biotypes of BVDV infection downregulate cytoplasmic and mitochondrial GPX4 via Nrf2-GPX4 pathway, thereby resulting in lethal lipid peroxidation and promoting ferroptosis. In parallel, BVDV can degrade ferritin heavy chain and mitochondrial ferritin via NCOA4-mediated ferritinophagy to promote the accumulation of Fe^2+^ and initiate ferroptosis. Importantly, CP BVDV-induced ferroptosis is tightly associated with serious damage of mitochondria and hyperactivation of inflammatory responses. In contrast, mild or unapparent damage of mitochondria and slight inflammatory responses were detected in NCP BVDV-infected cells. More importantly, different mitophagy pathways in response to mitochondria damage by both biotypes of BVDV are involved in inflammatory responses. Overall, this study is the first to show that mitochondria may play key roles in mediating ferroptosis and inflammatory responses induced by biotypes of BVDV *in vitro*.

**IMPORTANCE:**

Bovine viral diarrhea virus (BVDV) threatens a wide range of domestic and wild cattle population worldwide. BVDV causes great economic loss in cattle industry through its immunosuppression and persistent infection. Despite extensive research, the mechanism underlying the pathogenesis of BVDV remains elusive. Our data provide the first direct evidence that mitochondria-mediated ferroptosis and mitophagy are involved in inflammatory responses in both biotypes of BVDV-infected cells. Importantly, we demonstrate that the different degrees of injury of mitochondria and inflammatory responses may attribute to different mitophagy pathways induced by biotypes of BVDV. Overall, our findings uncover the interaction between BVDV infection and mitochondria-mediated ferroptosis, which shed novel light on the physiological impacts of ferroptosis on the pathogenesis of BVDV infection, and provide a promising therapeutic strategy to treat this important infectious disease with a worldwide distribution.

## INTRODUCTION

Bovine viral diarrhea virus (BVDV) is an important pathogen of cattle that can also infect a wide range of domestic and wide species including sheep, goats, deer, camelids, pigs, and wildlife animals (1, 2). BVDV infection causes immunologic suppression, enteritis, respiratory diseases, and reproductive disturbance in bovine (3). To date, underlying pathogenic mechanisms of BVDV are still not fully elucidated. The most unusual feature of BVDV is the existence of two biotypes in cell culture, such as cytopathic (CP) and non-cytopathic (NCP) effects (4, 5). The CP strains are the result of well-characterized genotypic changes that take place in a fatal form of BVDV, mucosal disease, which occurs in persistent infection animal where the NCP virus mutates to CP (6). Ample *in vitro* evidence implied that only NCP, but not CP, biotypes of BVDV are capable of establishing and maintaining persistent infection (7). This difference is associated with a different interaction of the two biotypes with the innate immune response against viral infection (7). NCP BVDV fails to induce interferon (IFN) type-I in various types of cultured cells, whereas infection with CP strain readily triggers IFN generation (4, 7).

Ferroptosis, an iron-dependent form of regulated necrosis, has emerged as a new cell death modality highly relevant to disease (8, 9). Ferroptosis results from the accumulation of cellular reactive oxygen species (ROS) that exceed the redox contents maintained by glutathione (GSH) and the phospholipid hydroperoxidases that use GSH as a substrate (8, 10). Cellular metabolism is essential for ferroptosis, presumably because lipid ROS is mainly generated from various steps of cellular metabolism (8). Mounting evidence has demonstrated that diverse cellular metabolism processes, including lipid metabolism and amino acid metabolism, contribute to ferroptosis (8, 9, 11). Despite the central role of mitochondria in oxidative metabolism, it remains unclear whether mitochondria play a central role in ferroptosis. In fact, ferroptosis is associated with dramatic morphological changes of mitochondria, including mitochondrial fragmentation and cristae enlargement, and some potent ferroptosis inhibitors appear to be exquisitely targeted to mitochondria (8, 10, 12). Mitochondrial ferritin (FtMt), an iron-storage protein located in mitochondria, has been shown to modulate cellular iron metabolism dramatically (13–15). Moreover, GPX4 is the most critical ferroptosis defense gene that encodes cytosolic, mitochondrial, and nucleolar isoforms (16). It has recently been demonstrated that mitochondrial GPX4 may play a critical role in pathogenesis of ferroptosis caused by atorvastatin (16). All these observations are consistent with the potential involvement of mitochondria in ferroptosis (17). However, there is also strong evidence arguing against a major role of mitochondria in ferroptosis (8). Taken together, the functional relevance of mitochondria in ferroptosis is still highly debatable.

Accumulating evidence has demonstrated the possible contribution of ferroptosis to viral replication and pathogenesis (18). Xu et al. reveal that herpes simplex virus 1-induced ferroptosis contributes to viral encephalitis (18). Furthermore, both enteroviruses and coronaviruses induce ferroptosis via ACSL4 to facilitate virus replication (19). However, whether BVDV induces ferroptosis in permissive cells remains elusive. More importantly, it is unclear whether and how ferroptosis functions in CP or NCP BVDV-caused distinct diseases, which therefore hinders the understanding of the physiological impact of ferroptosis on BVDV pathogenesis and the therapeutic potential of inhibiting ferroptosis in viral diseases.

In this study, we determine the involvement of ferroptosis and aberrant mitochondrial dynamics in Madin-Darby bovine kidney (MDBK) cells infected with CP or NCP BVDV biotype. Our data reveal that, although both biotypes of BVDV induced mitochondria-mediated ferroptosis at similar rate, serious damage of mitochondria and hyperactivation of inflammatory cytokine responses were detected in CP BVDV-infected cells, while mild or unapparent damage of mitochondria and slight inflammatory responses were detected in NCP BVDV-infected cells. Importantly, different mitophagy pathways induced by biotypes of BVDV are tightly associated with mitochondria damage and inflammatory responses. To the best of our knowledge, this study is the first to show that mitochondria may play key roles in mediating ferroptosis and inflammatory responses induced by biotypes of BVDV *in vitro*.

## RESULTS

### Both biotypes of BVDV induce ferroptosis in MDBK cells

**Fig 1:**
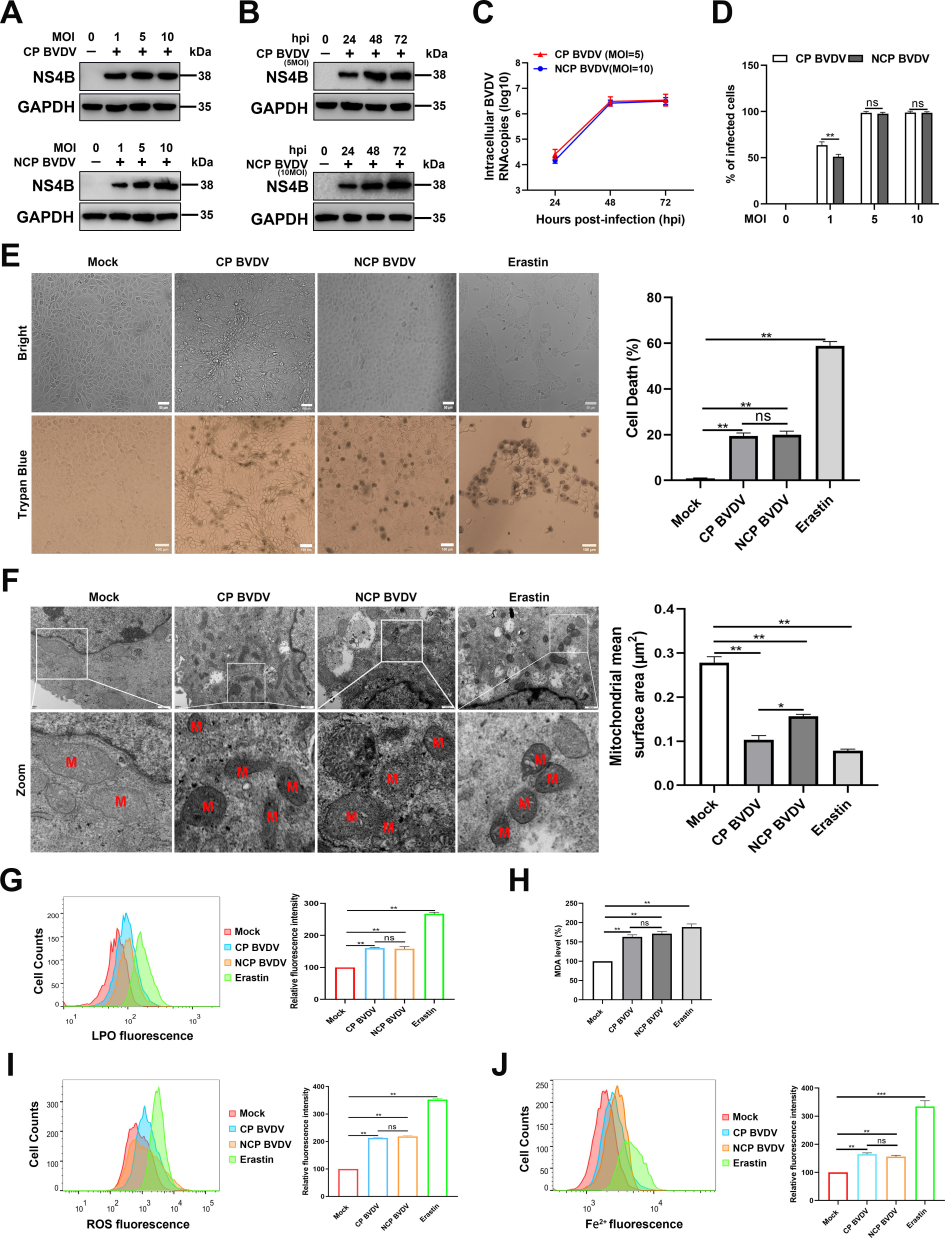
Both biotypes of BVDV induce ferroptosis in MDBK cells. (**A**) Western blot analysis of NS4B protein in mock-infected, BVDV-infected (MOI = 1, 5, 10), and mock-infected MDBK cells. Equal amounts of protein from BVDV- and mock-infected cells were separated using SDS-PAGE and transferred to PVDF membranes. The membranes were probed with NS4B antibody. (**B**) Western blot analysis of NS4B protein in mock-infected, CP BVDV-infected (MOI = 5), and NCP BVDV-infected (MOI = 10) MDBK cells from 24 to 72 hpi. (**C**) qRT-PCR analysis of BVDV N^pro^ gene in CP BVDV (MOI = 5)- and NCP BVDV (MOI = 10)-infected cells from 24 to 72 hpi. (**D**) Both biotypes of BVDV-infected cells (MOI = 1, 5, 10) were bound with BVDV NS4B antibody and then labeled with fluorophore-conjugated antibody or matched isotype controls and analyzed by flow cytometry at 48 hpi. (**E**) The percentage analysis of dead cells in mock-infected, CP BVDV (MOI = 5)-infected, and NCP BVDV (MOI = 10)-infected cells stained with trypan blue solution. Erastin (10 µM) as a positive control. Scale bar = 100 µm. (**F**) Transmission electron microscopy analysis of mitochondrial morphology in mock-infected, CP BVDV (MOI = 5)-infected, and NCP BVDV (MOI = 10)-infected cells at 48 hpi. Erastin (10 µM) as a positive control. Representative images of normal mitochondria or dysfunctional mitochondria are shown. Scale bar = 0.5 µm. (**G**) Flow cytometry analysis of intracellular lipid peroxidation levels in mock-infected, CP BVDV (MOI = 5)-infected, and NCP BVDV (MOI = 10)-infected cells stained with Lipofluo (5 µM) at 48 hpi. Then the mean fluorescence intensity of each cell was quantified. Erastin was used as the positive control. (**H**) Detection of malondialdehyde concentrations in mock-infected, CP BVDV (MOI = 5)-infected, and NCP BVDV (MOI = 10)-infected cells at 48 hpi. (**I**) Flow cytometry analysis of intracellular ROS levels in mock-infected, CP BVDV (MOI = 5)-, and NCP BVDV (MOI = 10)-infected cells stained with dichloro-ﬂuorescein diacetate (1 µM) at 48 hpi. Then the mean ROS fluorescence intensity of each cell was quantified. Erastin was used as the positive control. (**J**) Flow cytometry analysis of cellular ferrous iron in mock-infected, CP BVDV (MOI = 5)-, and NCP BVDV (MOI = 10)-infected cells stained with FerroOrange (1 µM) at 48 hpi. Then the mean FerroOrange fluorescence intensity of each cell was quantified. Erastin as a positive control. Data are given as means ± standard deviation from three independent experiments. *P* values were calculated using Student’s *t* test. An asterisk indicates a comparison with the indicated control. *, *P* < 0.05; **, *P* < 0.01; ns, not significant.

It has recently been demonstrated that various viral species can manipulate ferroptosis and facilitate their proliferation (18, 19). Nonetheless, whether ferroptosis plays an important role in virus infection remains highly controversial. Here, we evaluated whether BVDV, including CP and NCP BVDV biotypes, induces ferroptosis in MDBK cells. The ferroptosis inducer erastin was used as a positive control (10). We first determined the kinetics of virus replication in MDBK cells infected with CP or NCP BVDV. Western blot analysis revealed that an increased virus replication level of both biotypes of BVDV was detected in a dose-dependent manner at 48 hpi (Fig. 1A). However, the CP strain exhibited robust replication compared to the NCP strain at the same viral infection doses. In addition, both western blot and qRT-PCR analysis showed that the cells infected with CP [multiplicity of infection (MOI) = 5] or NCP BVDV (MOI = 10) exhibited similar viral replication properties (Fig. 1B and C). Furthermore, flow cytometry analysis showed that the percentage of infected cells following both biotypes of BVDV infection increased up to 100% with an MOI of either 5 or 10 at 48 hpi (Fig. 1D). Here, to avoid the effects of different viral replication levels on host cell responses, we conducted the downstream experiments for CP and NCP BVDV infection at MOI = 5 and MOI = 10, respectively. Trypan blue staining assay showed that both biotypes of BVDV induced cell death at similar rate, although CP BVDV induced cytopathic effect (CPE) in cultures at 48 hpi, while no significant CPE was detected in NCP BVDV-infected cells (Fig. 1E).

Moreover, we examined the morphological phenotype of mitochondria in MDBK cells infected with CP or NCP BVDV since a typical morphological feature of ferroptosis is the shrinkage of mitochondria (20). Transmission electron microscopy analysis revealed that both biotypes of BVDV-infected and erastin-treated cells displayed shrunk mitochondria with fewer cristae, increased mitochondrial membrane density, and decreased mitochondrial mean areas compared with those of mock-infected cells (Fig. 1F). Intriguingly, mild morphological changes of mitochondria were observed in NCP BVDV-infected cells compared to CP BVDV-infected and erastin-treated cells (Fig. 1F). Moreover, the increased cell death in both biotypes of BVDV-infected cells was accompanied by the increase in lipid peroxidation, including lipid ROS (Fig. 1G) and malondialdehyde (MDA) (Fig. 1H), another characteristic of ferroptosis. Lipid peroxidation results from the iron-dependent production of excessive ROS (21–23). Thus, we measured the intracellular levels of ROS and Fe^2+^ in BVDV-infected and erastin-treated cells using a ﬂuorometric intracellular ROS kit and an iron assay kit, respectively. Our results showed that both biotypes of BVDV induced the accumulation of ROS (Fig. 1I) and Fe^2+^ (Fig. 1J) in MDBK cells at similar rate.

Together, our ﬁndings indicate that both biotypes of BVDV induce ferroptosis in MDBK cells at similar rate.

### Ferroptosis inhibitor inhibits BVDV-induced ferroptosis in MDBK cells

**Fig 2:**
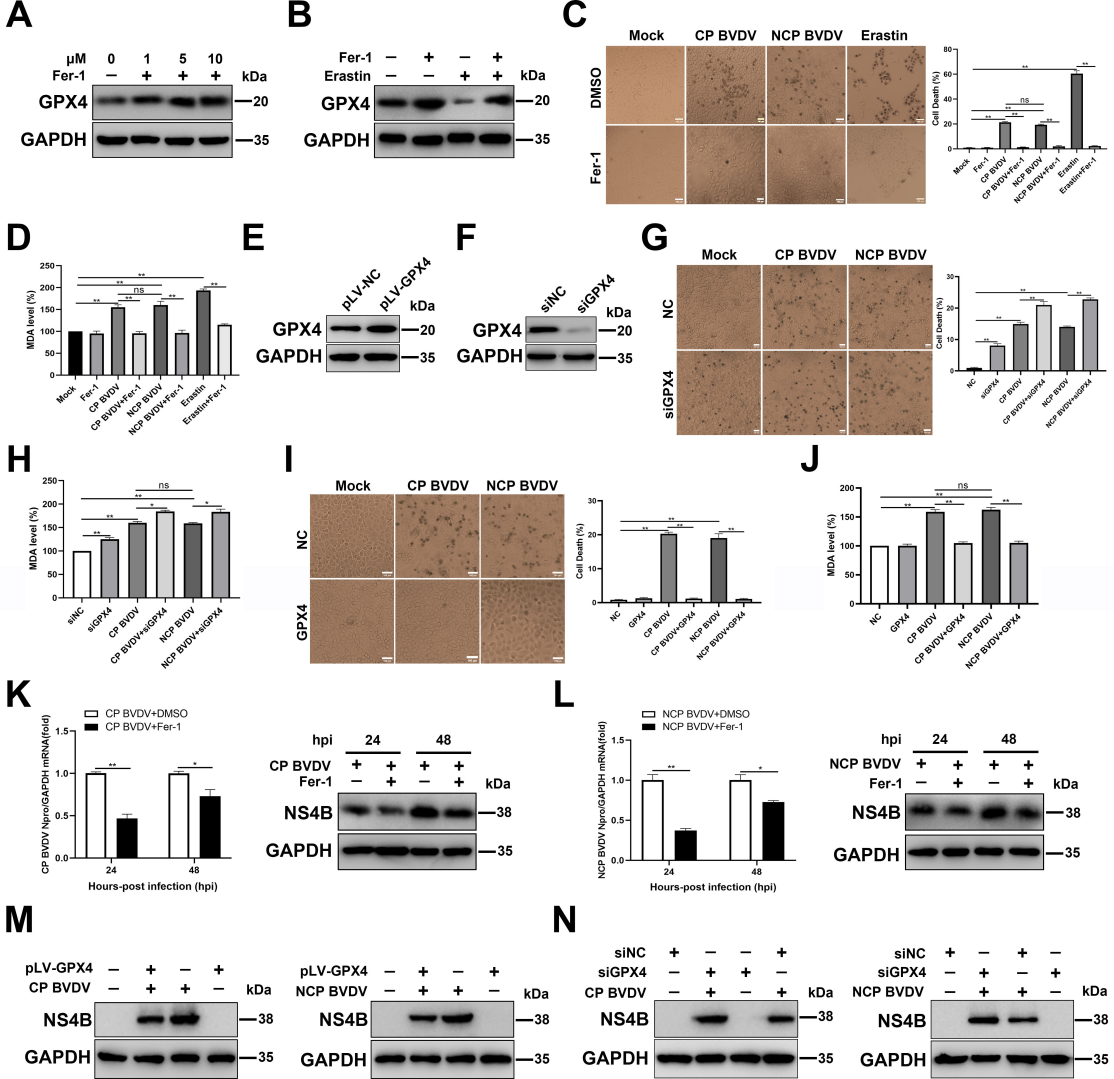
Ferroptosis inhibitor inhibits BVDV-induced ferroptosis in MDBK cells. (**A**) Western blot analysis of GPX4 protein in Fer-1-treated and mock-treated MDBK cells at 48 h. (**B**) Western blot analysis of GPX4 protein in erastin-treated and mock-treated MDBK cells with or without Fer-1 at 48 hpi. (**C**) The percentage analysis of dead cells in mock-infected, CP BVDV (MOI = 5)-, and NCP BVDV (MOI = 10)-infected cells stained with trypan blue solution with or without Fer-1 (10 µM) at 48 hpi. Erastin (10 µM) as a positive control. Scale bar = 100 µm. (**D**) Detection of MDA concentrations in mock-infected, CP BVDV (MOI = 5)-, and NCP BVDV (MOI = 10)-infected cells with or without Fer-1 at 48 hpi. Erastin (10 µM) as a positive control. (**E**) Establishment of MDBK cell lines stably overexpressing GPX4. Western blot analysis of GPX4 proteins in overexpressing GPX4 MDBK cells and NC MDBK cells was performed. (**F**) Western blot analysis of GPX4 expression in MDBK cells transfected with siGPX4 or siNC for 24 h. (**G**) The percentage analysis of dead cells in mock-infected, CP BVDV (MOI = 5)-, and NCP BVDV (MOI = 10)-infected cells stained with trypan blue solution with or without the transfection of siGPX4 or siNC at 48 hpi. Erastin (10 µM) as a positive control. Scale bar = 100 µm. (**H**) Detection of MDA concentrations in mock-infected, CP BVDV (MOI = 5)-, and NCP BVDV (MOI = 10)-infected cells with or without the transfection of siGPX4 or siNC at 48 hpi. (**I**) The percentage analysis of dead cells in mock-infected, CP BVDV (MOI = 5)-, and NCP BVDV (MOI = 10)-infected overexpressing GPX4 MDBK cells and NC MDBK cells stained with trypan blue solution at 48 hpi. Scale bar = 100 µm. (**J**) Detection of MDA concentrations in mock-infected, CP BVDV (MOI = 5)-, and NCP BVDV (MOI = 10)-infected overexpressing GPX4 MDBK cells and NC MDBK cells at 48 hpi. (**K**) qRT-PCR and western blot analysis of viral replication and progeny in CP BVDV-infected (MOI = 5) cells with or without Fer-1 at 24 and 48 hpi, respectively. (**L**) qRT-PCR and western blot analysis of viral replication and progeny in NCP BVDV-infected (MOI = 10) cells with or without Fer-1 at 24 and 48 hpi, respectively. (**M**) Western blot analysis of NS4B proteins in overexpressing GPX4 MDBK cells and NC MDBK cells infected with CP BVDV (MOI = 5) and NCP BVDV (MOI = 10) at 48 hpi. (**N**) Western blot analysis of NS4B protein in mock-infected, CP BVDV (MOI = 5)-, and NCP BVDV (MOI = 10)-infected cells with or without the transfection of siGPX4 or siNC at 48 hpi. Data are given as means ± standard deviation from three independent experiments. *P* values were calculated using Student’s *t* test. An asterisk indicates a comparison with the indicated control. *, *P* < 0.05; **, *P* < 0.01; ns, not significant.

Having shown that BVDV induced cell death through ferroptosis, we next sought to determine whether the inhibition of ferroptosis can protect against BVDV-induced cell death and lipid peroxidation. First, we treated cells with ferroptosis inhibitor Fer-1, a potent ferroptosis inhibitor that has been reported to prevent iron overload and ROS accumulation (24). Our data showed that the expression levels of GPX4, a crucial factor in ferroptosis pathway responsible for lipid ROS clearance (25), increased gradually in a dose-dependent manner after Fer-1 treatment (Fig. 2A). Furthermore, Fer-1 treatment abrogated decreased GPX4 levels in erastin-treated cells compared to untreated cells (Fig. 2B), confirming that Fer-1 treatment inhibited ferroptosis in MDBK cells. Subsequently, both biotypes of BVDV-infected and erastin-treated cells were incubated with vehicle or Fer-1. Compared to control cells, Fer-1 treatment rescued the cell death (Fig. 2C) and inhibited MDA levels (Fig. 2D) in BVDV-infected and erastin-treated cells. To further verify the involvement of ferroptosis during BVDV infection, we transfected cells with small interfering RNA (siRNA) targeting GPX4, or pLV-GPX4, or respective control, followed by BVDV infection. Western blot analysis showed that transfection of pLV-GPX4 plasmid significantly overexpressed GPX4 compared to control cells (Fig. 2E), while siRNA against GPX4 effectively inhibited GPX4 expression (Fig. 2F). It seems that GPX4 knockdown induced more serious cell death (Fig. 2G) and increased MDA levels (Fig. 2H) in either BVDV-infected or mock-infected cells compared to NC-transfected cells. In contrast, GPX4 overexpression rescued cell death (Fig. 2I) and decreased MDA levels (Fig. 2J) in either BVDV-infected or mock-infected cells compared to the respective control cells.

Recent studies have shown that viruses-induced ferroptosis contribute to virus replication (18, 19). Moreover, BVDV inducing the accumulation of ROS can enhance BVDV replication levels (26). Thus, we speculate that intracellular ROS production and, subsequently, the induction of ferroptosis are important for BVDV replication. To test this possibility, we examined BVDV replication in infected cells in the presence or absence of Fer-1. Our data showed that Fer-1 treatment signiﬁcantly suppressed CP (Fig. 2K) and NCP BVDV (Fig. 2L) replication levels in cells. Furthermore, GPX4 overexpression inhibited viral levels (Fig. 2M), while GPX4 knockdown enhanced viral levels in both biotypes of BVDV-infected cells (Fig. 2N) compared to respective control cells.

Collectively, these results demonstrate that both biotypes of BVDV-induced ferroptosis enhanced virus replication.

### BVDV induces the accumulation of lipid peroxidation in mitochondria

**Fig 3:**
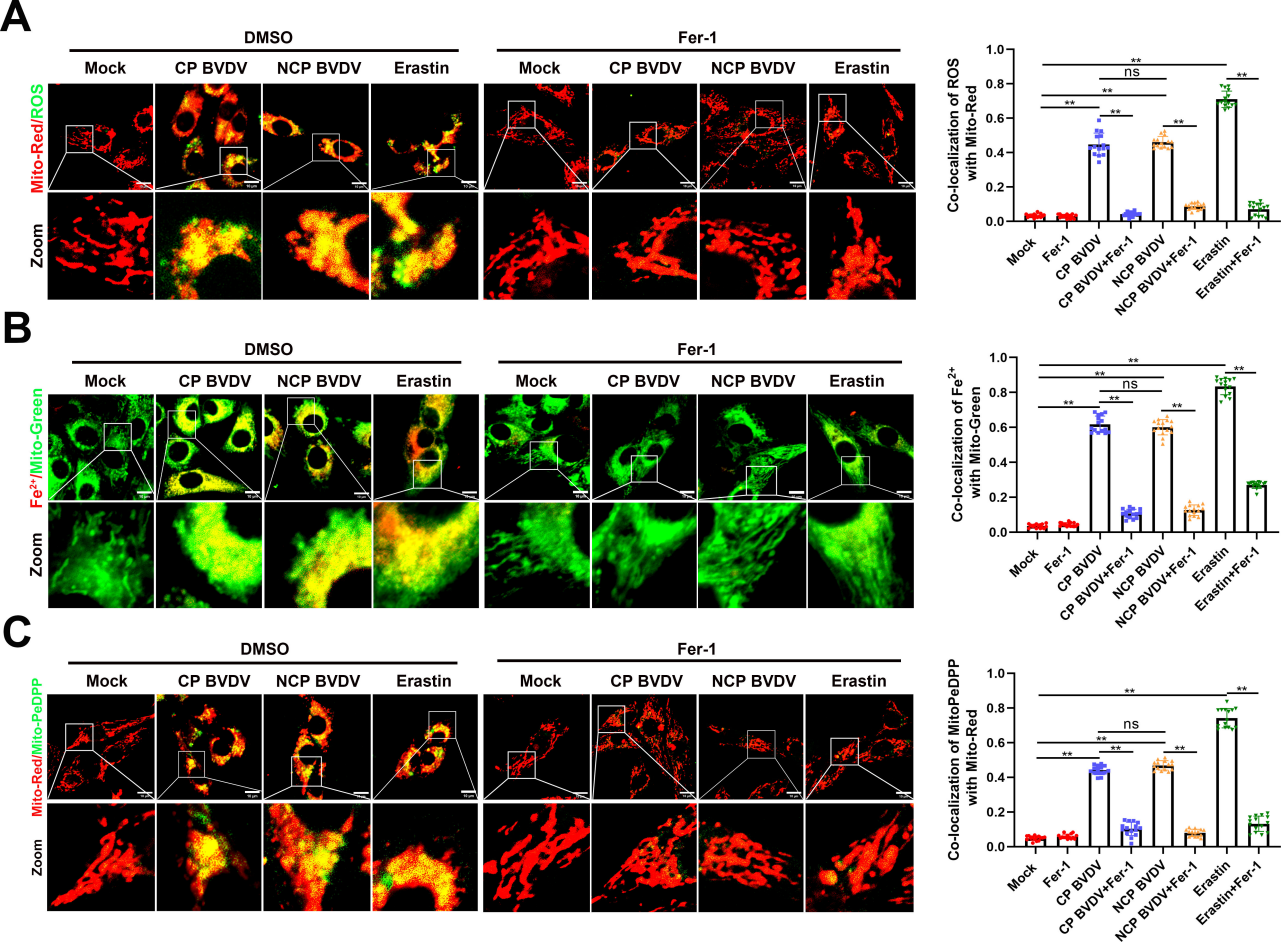
BVDV induces the accumulation of lipid peroxidation in mitochondria. (**A**) IFA analysis of the intracellular ROS levels in mitochondria from mock-infected, CP BVDV (MOI = 5)-, and NCP BVDV (MOI = 10)-infected cells stained with DCFH-DA (1 µM) with or without Fer-1 at 48 hpi. Erastin (10 µM) as a positive control. Scale bar = 10 µm. (**B**) IFA analysis of the cellular ferrous iron levels in mitochondria from mock-infected, CP BVDV (MOI = 5)-, and NCP BVDV (MOI = 10)-infected cells stained with FerroOrange (1 µM) with or without Fer-1 at 48 hpi. Erastin (10 µM) as a positive control. Scale bar = 10 µm. (**C**) IFA analysis of the mitochondrial lipid peroxidation levels in mock-infected, CP BVDV (MOI = 5)-, and NCP BVDV (MOI = 10)-infected cells stained with MitoPeDPP (1 µM) with or without Fer-1 at 48 hpi. Erastin (10 µM) as a positive control. Scale bar = 10 µm. Data are given as means ± standard deviation from three independent experiments. *P* values were calculated using Student’s *t* test. An asterisk indicates a comparison with the indicated control. *, *P* < 0.05; **, *P* < 0.01; ns, not significant.

Whether the mitochondria are an important component in ferroptosis induction remains highly controversial (8). To analyze the role of mitochondria in BVDV-induced ferroptosis, mitochondrial content of ROS, Fe^2+^, and lipid peroxidation was detected in both biotypes of BVDV-infected cells in the presence or absence of Fer-1. A dichloro-ﬂuorescein diacetate (DCFH-DA) and an iron probe (FerroOrange) were used to detect the signals of ROS and Fe^2+^ in mitochondria, respectively (27). MitoPeDPP was used to examine the mitochondrial lipid peroxidation (16). Immunofluorescence analysis showed that both biotypes of BVDV infection induced strong accumulation of ROS (Fig. 3A), Fe^2+^ (Fig. 3B), and lipid peroxidation (Fig. 3C) in mitochondria. However, Fer-1 treatment significantly suppressed the accumulation of ROS (Fig. 3A), Fe^2+^ (Fig. 3B), and lipid peroxidation (Fig. 3C) in both biotypes of BVDV-infected mitochondria. Collectively, the ferroptosis indicator changed signiﬁcantly on mitochondria in response to CP or NCP BVDV infection, suggesting that mitochondria may play a critical role in BVDV-induced ferroptosis.

### BVDV downregulating mitochondrial GPX4 via inhibiting the protein levels of Nrf2

**Fig 4:**
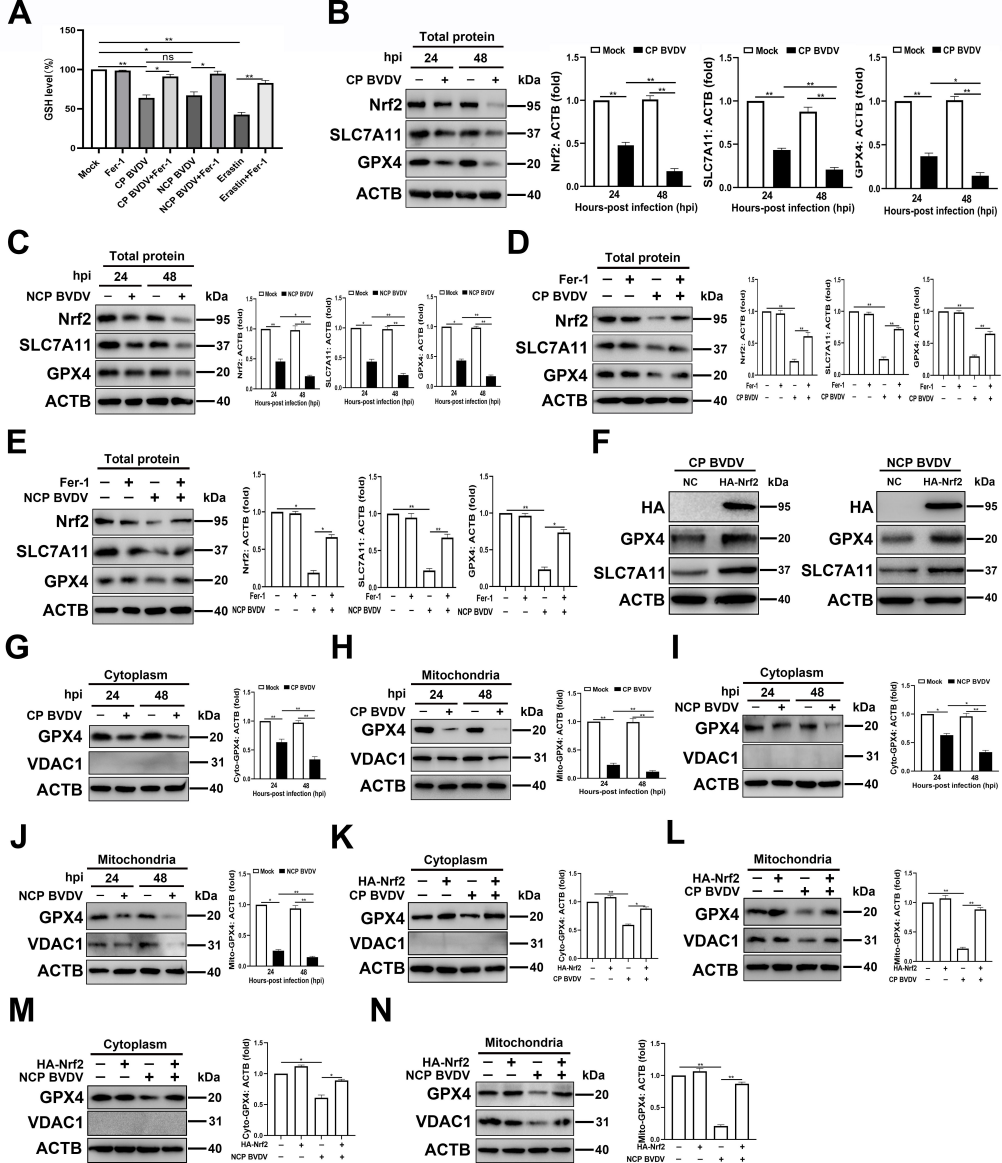
BVDV downregulating mitochondrial GPX4 via inhibiting the protein levels of Nrf2. (**A**) Detection of intracellular GSH concentrations in mock-infected, CP BVDV (MOI = 5)-, and NCP BVDV (MOI = 10)-infected cells or erastin-treated cells with or without Fer-1 at 48 hpi. (**B and C**) Western blot analysis of Nrf2, SLC7A11, and GPX4 proteins in MDBK cells infected with CP BVDV (MOI = 5) (**B**) and NCP BVDV (MOI = 10) (**C**) at 24 and 48 hpi, respectively. (**D and E**) Western blot analysis of Nrf2, SLC7A11, and GPX4 proteins in MDBK cells infected with CP BVDV (MOI = 5) (**D**) and NCP BVDV (MOI = 10) (**E**) with or without Fer-1 at 48 hpi, respectively. (**F**) Establishment of MDBK cell lines stably overexpressing HA-Nrf2. Western blot analysis of GPX4 and SLC7A11 proteins in overexpressing HA-Nrf2 MDBK cells and NC MDBK cells infected with CP BVDV (MOI = 5) and NCP BVDV (MOI = 10) at 48 hpi, respectively. (**G and H**) Western blot analysis of GPX4 and VDAC1 proteins in cytoplasmic fractions (**G**) and mitochondrial fractions (**H**) from MDBK cells infected with CP BVDV (MOI = 5) at 24 and 48 hpi, respectively. (**I and J**) Western blot analysis of GPX4 and VDAC1 proteins in cytoplasmic fractions (**I**) and mitochondrial fractions (**J**) from MDBK cells infected with NCP BVDV (MOI = 10) at 24 and 48 hpi, respectively. (**K and L**) Western blot analysis of GPX4 and VDAC1 proteins in cytoplasmic fractions (**K**) and mitochondrial fractions (**L**) from MDBK cell lines stably overexpressing HA-Nrf2 infected with CP BVDV (MOI = 5) at 48 hpi, respectively. (**M and N**) Western blot analysis of GPX4 and VDAC1 proteins in cytoplasmic fractions (**M**) and mitochondrial fractions (**N**) from MDBK cell lines stably overexpressing HA-Nrf2 infected with NCP BVDV (MOI = 10) at 48 hpi, respectively. Data are given as means ± standard deviation from three independent experiments. *P* values were calculated using Student’s *t* test. An asterisk indicates a comparison with the indicated control. *, *P* < 0.05; **, *P* < 0.01; ns, not significant.

One of the key initial signals proposed to trigger ferroptosis is inhibition of system X_C_^−^ antiporter, which is a heterodimer made of solute carrier (SLC) family 7 member (SLC7A11) and SLC3A2 (27). This antiporter functions to import cystine into the cell where it is reduced to cysteine and used for glutathione synthesis (28). GSH works to reduce ROS in the cell via the function of GSH peroxidases such as GPX4. GPX4 mediates the process of GSH oxidization to glutathione disulfide (GSSG) for scavenging excessive ROS (25). Therefore, after identifying that BVDV induced excessive ROS production and ferroptosis in MDBK cells, we sought to determine the GSH levels in BVDV-infected cells using a GSSG/GSH quantification kit. Our data showed that GSH levels were decreased signiﬁcantly in both CP and NCP BVDV-infected cells compared to mock-infected cells (Fig. 4A). Furthermore, Fer-1 treatment can rescue biosynthesis of GSH upon BVDV infection, suggesting the biosynthesis of GSH is interrupted in BVDV-infected cells, and this process contributes to excessive ROS accumulation and, subsequently, ferroptosis (Fig. 4A).

Nrf2 is identified as an important regulator for the expression of a series of antioxidant genes, including GSH homeostasis, and GPX4 activity (29). To explore the role of Nrf2 in BVDV-induced ferroptosis, we focused on the antioxidative genes implicated in negatively regulating ferroptosis, including SLC7A11 and GPX4 (30). Our data showed that both biotypes of BVDV infection suppressed the expression of Nrf2, SLC7A11, and GPX4 in MDBK cells compared to mock-infected cells in a post infection time-dependent manner (Fig. 4B and C). Fer-1 treatment rescued the decreased expression of Nrf2, SLC7A11, and GPX4 in both biotypes of BVDV-infected cells (Fig. 4D and E). Furthermore, overexpression of Nrf2 counterbalanced the inhibitory effect of BVDV infection on the protein level of endogenous SLC7A11 and GPX4 (Fig. 4F). To determine the expression of mitochondrial and cytoplasmic GPX4 upon BVDV infection, as well as the role of Nrf2 in GPX4 expression, purified mitochondrial fractions and cytoplasmic fractions from CP- or NCP BVDV-infected cells were detected by western blot. No detectable expression of VDAC1 in the purified cytoplasmic fractions from BVDV- and mock-infected cells confirmed that there was no mitochondria contamination in cytoplasmic fractions (Fig. 4G, I, K, and M). Our data showed that GPX4 expression in BVDV-infected cells was decreased more significantly in mitochondria than that exists in cytoplasm compared to mock-infected cells (Fig. 4G through J). Overexpression of Nrf2 rescued the decreased GPX4 expression in both mitochondrial and cytoplasmic fractions in BVDV-infected cells (Fig. 4K through N).

Together, we conclude that both biotypes of BVDV-induced Nrf2 downregulation cause the loss of its antiferroptotic activity, which contributes to interrupting GSH biosynthesis and promoting ferroptosis.

### BVDV-induced NCOA4-mediated ferritinophagy contributes to the degradation of FTH and FtMt

Iron storage proteins, including FTH as well as FtMt, have a signiﬁcant role in modulating ferroptosis through its ferroxidase activity (31). To explore the role of FTH and FtMt in BVDV-induced ferroptosis, we first examine the expression of FtMt and FTH in BVDV-infected cells. Western blot analysis showed that both FTH and FtMt were decreased by BVDV in a time-dependent manner in MDBK cells (Fig. 6A and B), coinciding with the increases in Fe^2+^ production (Fig. 1J).

**Fig 5:**
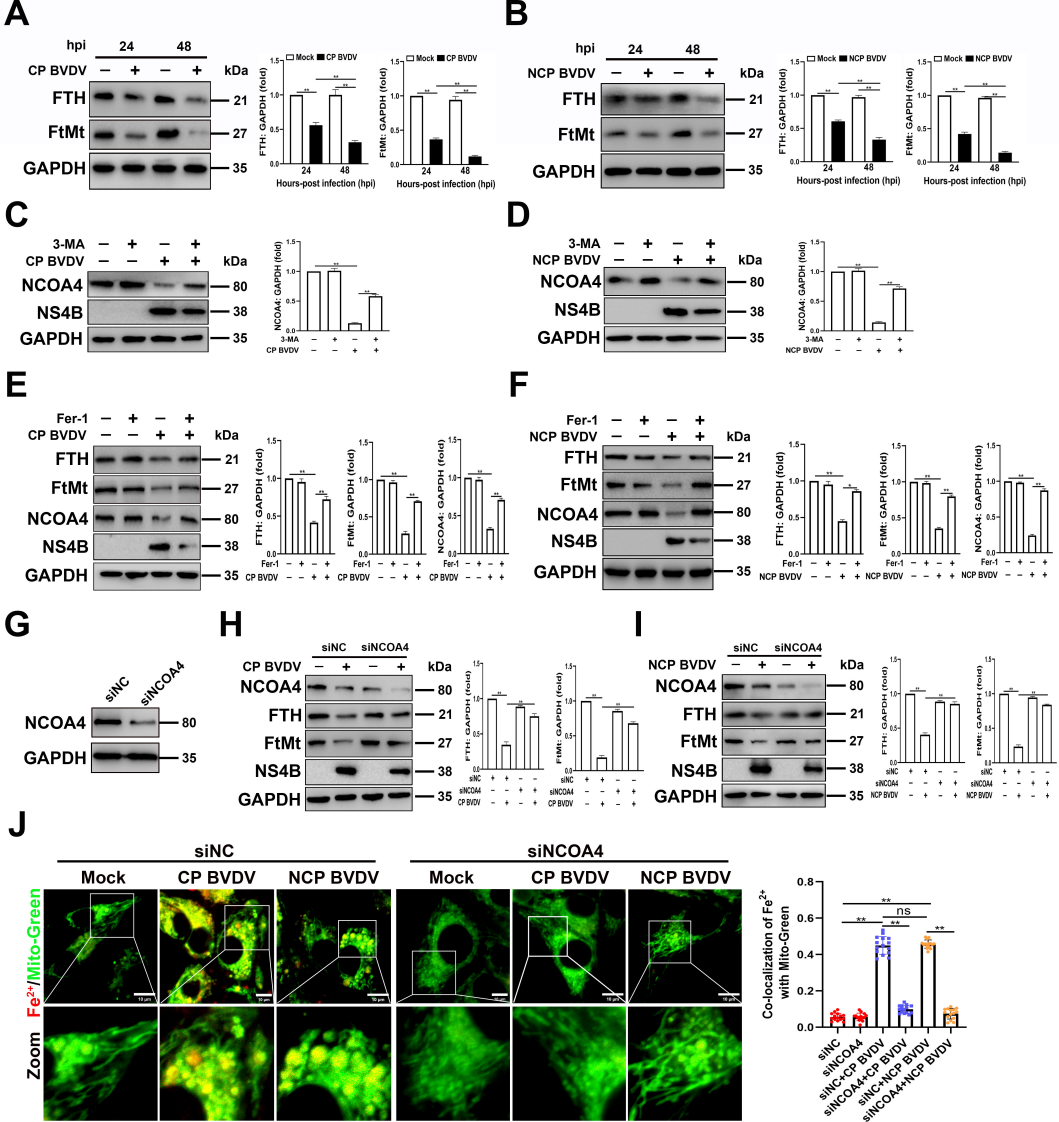
BVDV-induced NCOA4-mediated ferritinophagy contributes to the degradation of FTH and FtMt. (**A and B**) Western blot analysis of FtMt, FTH, and NS4B proteins in MDBK cells infected with CP BVDV (MOI = 5) (**A**) and NCP BVDV (MOI = 10) (**B**) at 24 and 48 hpi, respectively. (**C and D**) Western blot analysis of NCOA4 and NS4B proteins in MDBK cells infected with CP BVDV (MOI = 5) (**C**) and NCP BVDV (MOI = 10) (**D**) with or without 3-methyladenine (5 mM) at 48 hpi, respectively. (**E and F**) Western blot analysis of FtMt, NCOA4, FTH, and NS4B proteins in MDBK cells infected with CP BVDV (MOI = 5) (**E**) and NCP BVDV (MOI = 10) (**F**) with or without Fer-1 at 48 hpi, respectively. (**G**) Western blot analysis of NCOA4 expression in MDBK cells transfected with siNCOA4 or siNC for 24 h. (**H and I**) Western blot analysis of NCOA4, FTH, FtMt, and NS4B proteins in CP BVDV (MOI = 5)- (**H**) and NCP BVDV (MOI = 10)-infected cells (**I**) with or without the transfection of siNCOA4 or siNC at 48 hpi, respectively. (**J**) IFA analysis of Fe^2+^ and mitochondria in CP BVDV (MOI = 5)- and NCP BVDV (MOI = 10)-infected cells with or without the transfection of siNCOA4 or siNC at 48 hpi. Scale bar = 10 µm. Data are given as means ± standard deviation from three independent experiments. *P* values were calculated using Student’s *t* test. An asterisk indicates a comparison with the indicated control. *, *P* < 0.05; **, *P* < 0.01; ns, not significant.

**Fig 6:**
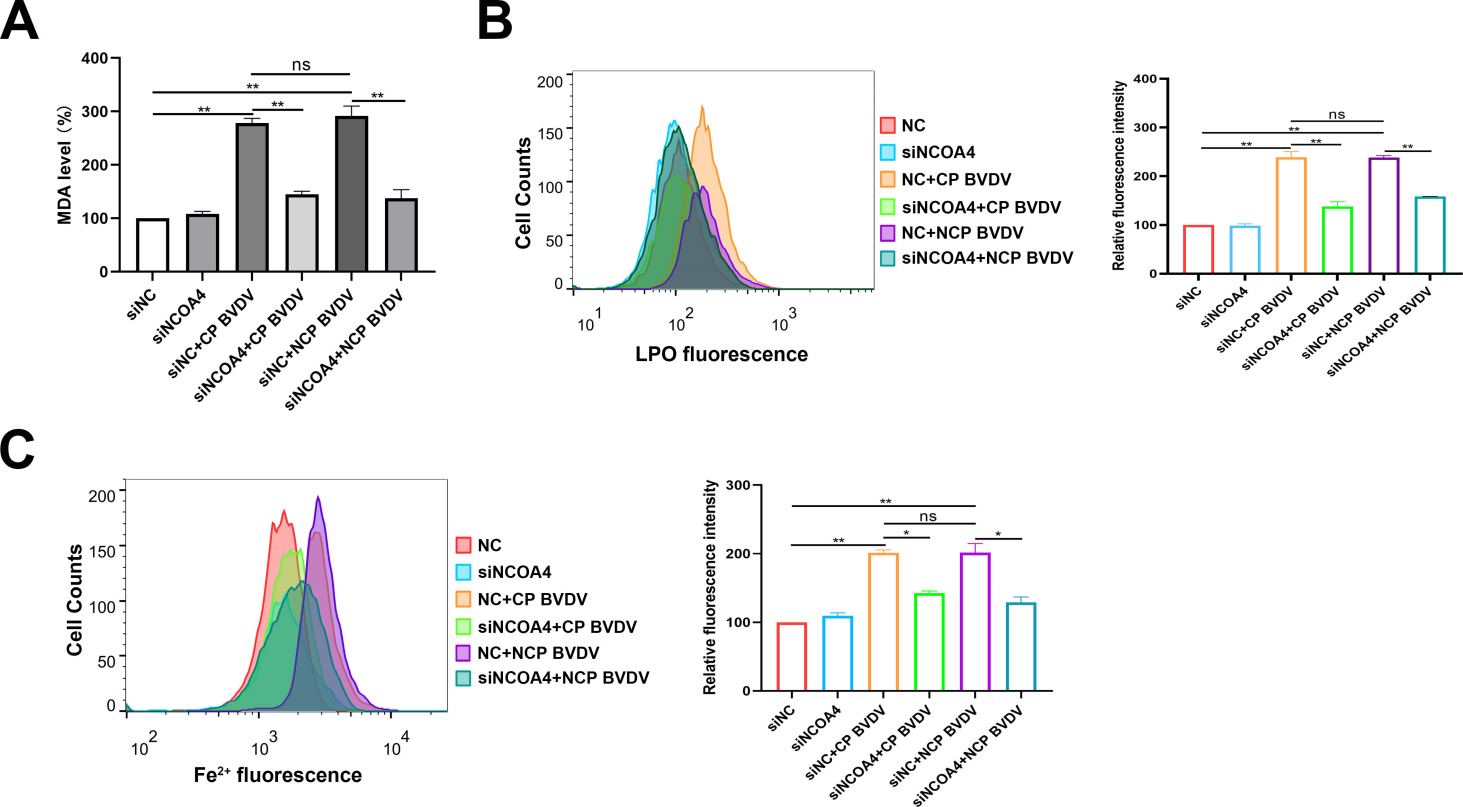
BVDV-induced ferritinophagy initiates ferroptosis. (**A**) Detection of MDA concentrations in mock-infected, CP BVDV (MOI = 5)-, and NCP BVDV (MOI = 10)-infected cells with or without the transfection of siNCOA4 or siNC at 48 hpi. (**B**) Flow cytometry analysis of intracellular lipid peroxidation levels in mock-infected, CP BVDV (MOI = 5)-, and NCP BVDV (MOI = 10)-infected cells stained with Lipofluo with or without the transfection of siNCOA4 or siNC at 48 hpi. Then the mean fluorescence intensity of each cell was quantified. Erastin was used as the positive control. (**C**) Flow cytometry analysis of cellular ferrous iron in mock-infected, CP BVDV (MOI = 5)-, and NCP BVDV (MOI = 10)-infected cells stained with FerroOrange with or without the transfection of siNCOA4 or siNC at 48 hpi. Then the mean FerroOrange fluorescence intensity of each cell was quantified. Data are given as means ± standard deviation from three independent experiments. *P* values were calculated using Student’s *t* test. An asterisk indicates a comparison with the indicated control. *, *P* < 0.05; **, *P* < 0.01; ns, not significant.

Iron release from ferritins is regulated by a process known as ferritinophagy (32). Therefore, after identifying that BVDV inhibits the expression of FTH and FtMt in MDBK cells, we sought to examine the activation of ferritinophagy by detecting the NCOA4 level, a critical mediator for ferritinophagy activation, in BVDV-infected cells. Western blot analysis showed that NCOA4 levels were significantly reduced in both biotypes of BVDV-infected cells compared to mock-infected cells. However, NCOA4 degradation was reversed after treatment with autophagy inhibitor 3-methyladenine (3-MA) (Fig. 6C and D). Moreover, 3-MA treatment reduced viral levels in both biotypes of BVDV-infected cells (Fig. 6C and D). Then, we detect the activation of ferritinophagy on the expression of FTH and FtMt in BVDV-infected cells treated with Fer-1. Our data showed that the protein levels of NCOA4 were decreased in response to BVDV infection in the absence of Fer-1, which was accompanied with decreased expression of FTH and FtMt in MDBK cells. However, Fer-1 treatment abolished the change of the expression of NCOA4, FTH, and FtMt in BVDV-infected cells compared to mock-infected cells (Fig. 6E and F). To conﬁrm the role of NCOA4 in ferritins-mediated ferroptosis during BVDV infection, MDBK cells were transfected with an siRNA against *NCOA4* (siNCOA4) or a non-targeting control siRNA (siNC) for 24 h. NCOA4 protein expression was effectively inhibited by transfection of siRNA (Fig. 5G). Knocking down NCOA4 strongly enhanced FTH and FtMt expression in BVDV-infected cells (Fig. 6H and I), coinciding with the decrease of Fe^2+^ in cytoplasm and mitochondria, respectively, as observed by IFA (Fig. 6J).

Together, our findings indicate that BVDV-induced NCOA4-mediated ferritinophagy contributes to the degradation of FTH and FtMt and, subsequently, the accumulation of Fe^2+^ in cytoplasm and mitochondria, respectively.

### BVDV-induced ferritinophagy initiates ferroptosis

**Fig 7:**
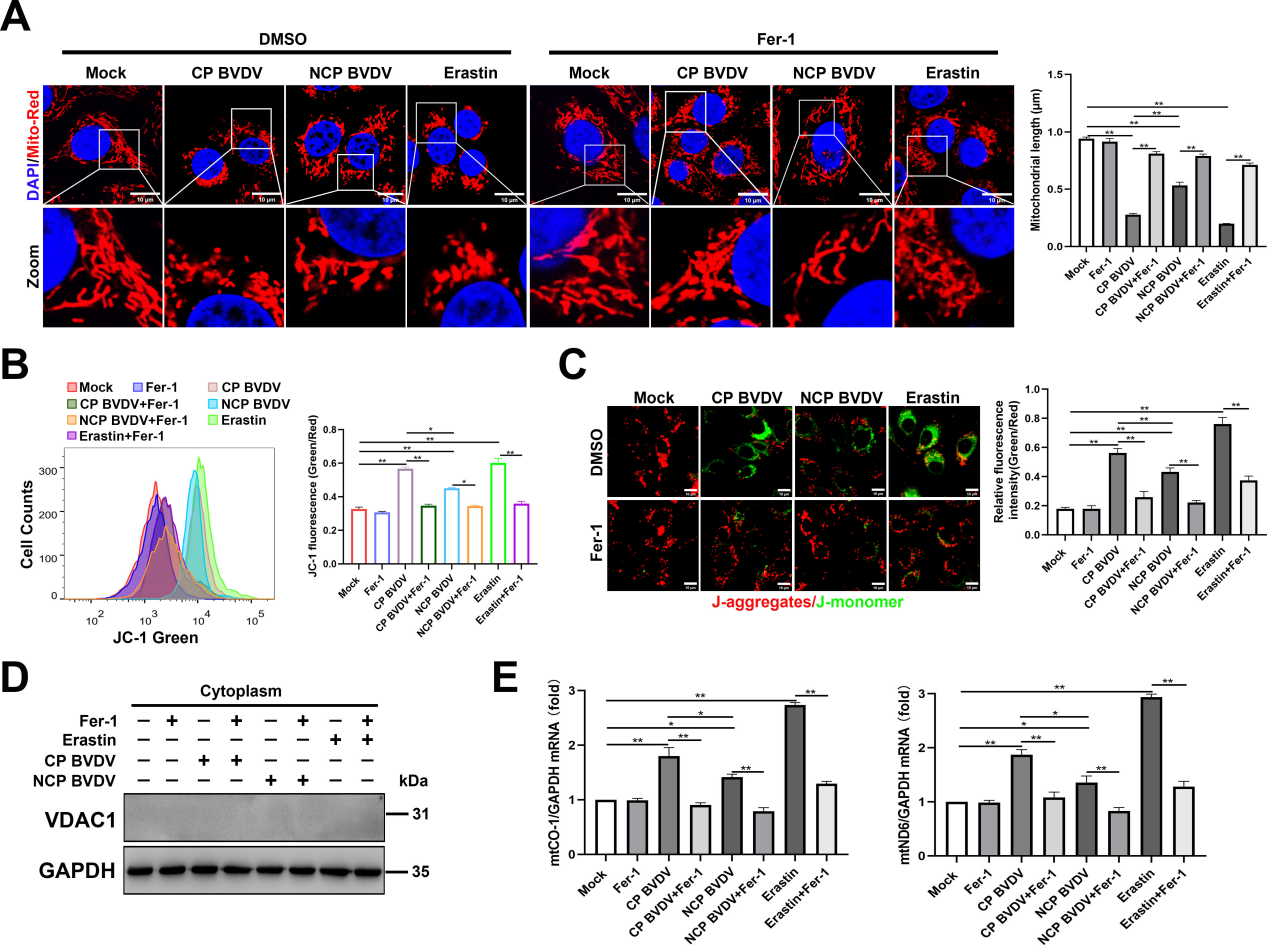
BVDV-induced ferroptosis caused the impairment of mitochondria. (**A**) IFA analysis of mitochondrial morphology labeled by Mito Tracker Red in mock-infected, CP BVDV (MOI = 5)-, and NCP BVDV (MOI = 10)-infected cells or erastin-treated cells with or without Fer-1 at 48 hpi. Scale bar = 10 µm. (**B**) Flow cytometry analysis of mitochondrial membrane potential (MMP) in mock-infected, CP BVDV (MOI = 5)-, and NCP BVDV (MOI = 10)-infected cells stained with JC-1 with or without Fer-1 at 48 hpi, erastin as a positive control, MMP was determined by green/red ratio of mean fluorescence intensity. (**C**) IFA analysis of MMP in mock-infected, CP BVDV (MOI = 5)-, and NCP BVDV (MOI = 10)-infected cells stained with JC-1 with or without Fer-1 at 48 hpi. Erastin as a positive control. Scale bar = 10 µm. MMP was determined by green/red ratio of mean fluorescence intensity using Image J software (Version 1.53t) (NIH). (**D**) Western blot analysis of VDAC1 protein in cytoplasmic fractions from MDBK cells infected with CP BVDV (MOI = 5) and NCP BVDV (MOI = 10) or treated with erastin in the presence or absence of Fer-1 at 48 hpi. (**E**) qRT-PCR analysis of mtCO1 and mtND6 genes in mock-infected, CP BVDV (MOI = 5), and NCP BVDV (MOI = 10) in the presence or absence of Fer-1 at 48 hpi. Data are given as means ± standard deviation from three independent experiments. *P* values were calculated using Student’s *t* test. An asterisk indicates a comparison with the indicated control. *, *P* < 0.05; **, *P* < 0.01; ns, not significant.

After identifying that BVDV-induced ferritinophagy degrades FTH and FtMt in MDBK cells, we next investigate the role of ferritinophagy in the process of ferroptosis. We examined the levels of MDA, lipid peroxides, and Fe^2+^ after NCOA4 knockdown. Our findings indicated that knocking down endogenous NCOA4 suppressed the induction of MDA (Fig. 7A) and lipid peroxides (Fig. 7B), as well as Fe^2+^ levels (Fig. 7C) in both biotypes of BVDV-infected cells. These findings indicate that BVDV accumulates ferrous iron via a decrease in ferritin through ferritinophagy and induces ferroptosis in MDBK cells.

### BVDV-induced ferroptosis caused the impairment of mitochondria

Ferroptosis is associated with dramatic morphological changes of mitochondria, including mitochondrial fragmentation and cristae enlargement (8). Our IFA analysis showed that fragmented mitochondrial morphology was observed in both biotypes of BVDV-infected cells compared to the typical tubular mitochondria in mock-infected cells (Fig. 5A). However, Fer-1 treatment remarkably ameliorated both biotypes of BVDV-induced mitochondrial abnormality (Fig. 5A). Intriguingly, a more extensive fragmentation of mitochondria following CP BVDV infection compared to that in NCP BVDV was observed (Fig. 5A). Next, we analyzed whether mitochondria lost their mitochondrial membrane potential (MMP) by JC-1 probe in BVDV-infected cells. The flow cytometry analysis showed that there was a significant MMP reduction in both biotypes of BVDV and erastin treatment groups compared to mock-infected cells; however, a more MMP reduction in CP BVDV-infected cells compared to NCP BVDV-infected cells was detected (Fig. 5B). Fer-1 treatment remarkably ameliorated BVDV-induced MMP reduction (Fig. 5B). Similar results were obtained by the fluorescence analysis (Fig. 5C). Mock-infected cells exhibited normal MMP, characterized by increased red fluorescence (JC-1 aggregate) with a lesser green fluorescence (JC-1 monomer). In contrast, both biotypes of BVDV-infected and erastin-treated cells exhibited decreased MMP, as shown by increased shift in red to green fluorescence (Fig. 5C). Intriguingly, a more MMP reduction in CP BVDV-infected and erastin-treated cells compared to NCP BVDV-infected cells was detected (Fig. 5C). Again, Fer-1 treatment rescued BVDV-induced MMP reduction (Fig. 5C). The reduction of MMP can cause leakage of many mitochondrial contents, including mitochondrial DNA (mtDNA), which are likely to participate in innate immune responses (33). Since both biotypes of BVDV infection impaired mitochondria as observed in this study, we performed qPCR assays to determine the levels of cytosolic mtDNA following CP or NCP BVDV infection. No detectable VDAC1 expression in purified cytoplasmic fractions from the cells confirmed there was no mitochondria contamination in cytoplasmic fractions (Fig. 5D). Our data revealed that both biotypes of BVDV infection enhanced the levels of cytosolic mtDNA (*mt-CO1* and *mt-ND6*) compared to mock-infected cells, of note, CP BVDV infection induced more cytosolic mtDNA levels as compared with NCP BVDV-infected cells (Fig. 5E), which may attribute to the severe damaged mitochondria caused by CP BVDV infection. However, treatment of cells with Fer-1 inhibited cytosolic mtDNA levels in both biotypes of BVDV-infected cells compared to untreated control cells (Fig. 5E).

Together, these findings reveal that ferroptosis induced by both biotypes of BVDV, in particular CP BVDV, causes mitochondria damage and leakage of mitochondrial contents.

### Mitophagy is involved in BVDV-induced ferroptosis-mediated inflammatory responses

**Fig 8:**
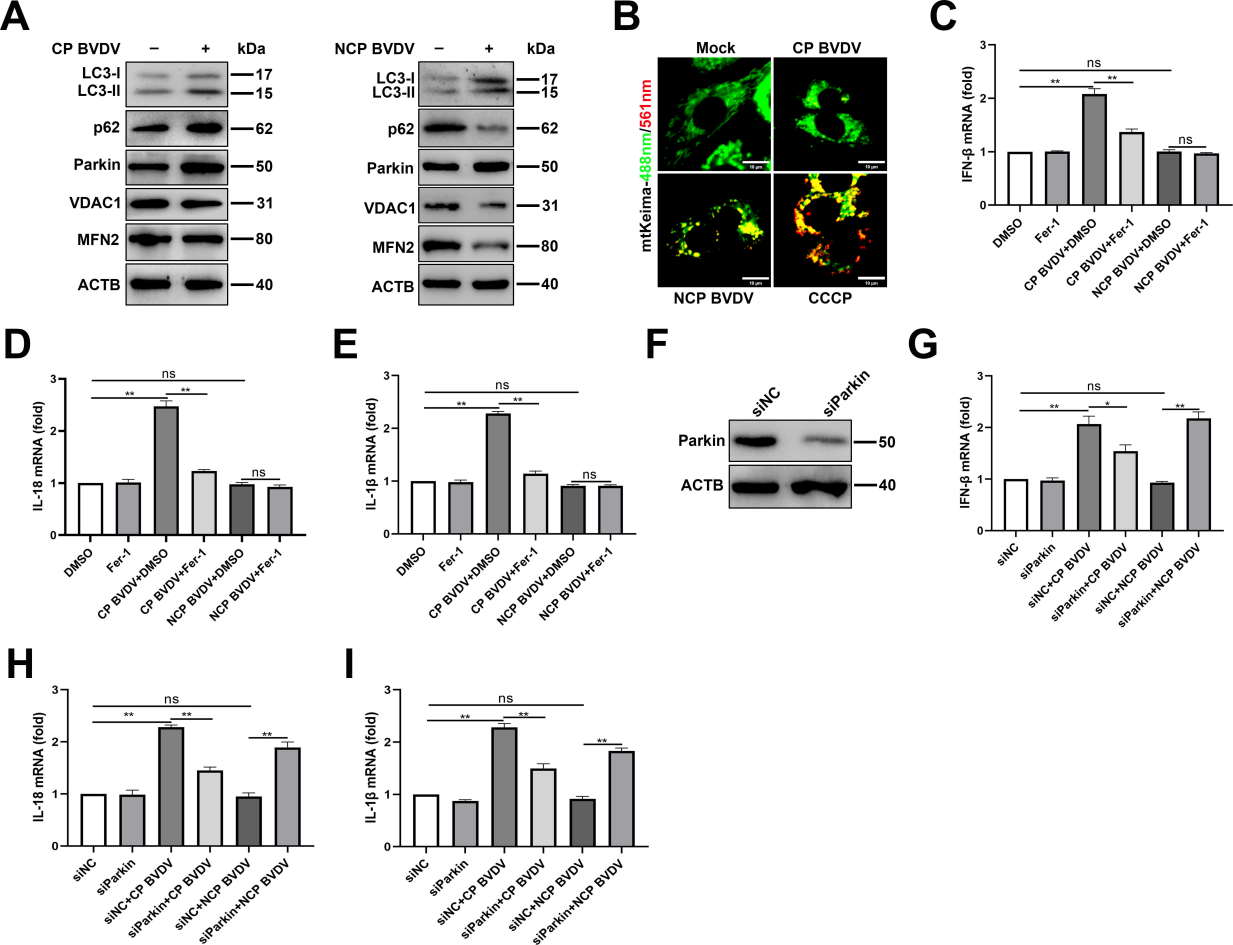
Mitophagy is involved in BVDV-induced ferroptosis-mediated inflammatory responses. (**A**) Western blot analysis of LC3-II, p62, Parkin, VDAC1, and MFN2 proteins in mock-infected, CP BVDV (MOI = 5)-, and NCP BVDV (MOI = 10)-infected cells at 48 hpi. (**B**) IFA analysis of mt-Keima localization in cells infected with CP BVDV (MOI = 5) and NCP BVDV (MOI = 10) at 48 hpi. Scale bar = 10 µm. (**C–E**) qPCR analysis of IFN-β (**C**), IL-18 (**D**), and IL-1β (**E**) genes in mock-infected, CP BVDV (MOI = 5)-, and NCP BVDV (MOI = 10)-infected cells with or without Fer-1 at 48 hpi. (**F**) Western blot analysis of Parkin expression in MDBK cells transfected with siParkin or siNC for 24 h. (**G–I**) qPCR analysis of IFN-β (**G**), IL-18 (**H**), and IL-1β (**I**) genes in mock-infected, CP BVDV (MOI = 5)-, and NCP BVDV (MOI = 10)-infected cells with or without the transfection of siParkin or siNC at 48 hpi. Data are given as means ± standard deviation from three independent experiments. *P* values were calculated using Student’s *t* test. An asterisk indicates a comparison with the indicated control. *, *P* < 0.05; **, *P* < 0.01; ns, not significant.

The clearance of damaged mitochondria via mitophagy is crucial for establishing mitochondrial homeostasis (34, 35). After identifying that both biotypes of BVDV induce ferroptosis-mediated mitochondria damage, we determine the role of mitophagy during BVDV infection. To further examine this, we probed for mitophagy-related genes Parkin, LC3-II, and p62, as well as mitochondrial proteins MFN2 and VDAC1. An increased expression level of LC3-II and Parkin in both biotypes of BVDV groups was detected compared to mock-infected cells, which was accompanied with increased abundance of p62 in CP BVDV-infected cells and decreased p62 in NCP BVDV-infected cells compared to mock-infected cells (Fig. 8A), suggesting NCP BVDV infection induced complete mitophagy and CP BVDV induced defective mitophagy. Defective mitophagy is likely due to a blockade in mitophagosome maturation and/or its clearance. In fact, decreased VDAC1 and MFN2 expression in NCP BVDV-infected cells and no significant changes for VDAC1 and MFN2 expression in CP BVDV-infected cells compared to mock-infected cells were detected in this study (Fig. 8A). To further monitor mitophagy flux, we expressed mt-Keima, a pH-sensitive fluorescent protein which presents green fluorescence under neutral environments such as normal mitochondria and presents red fluorescence under acidic pH when mitochondria are engulfed by lysosomes during mitophagy (36). A far greater number of red dots appeared in NCP BVDV-infected and CCCP treatment, a known inducer of mitophagy, cells transfected with mt-Keima (Fig. 8B), while more green dots appeared in CP BVDV-infected and mock-infected cells (Fig. 8B), suggesting that an increased accumulation of mitophagosomes in response to both biotypes of BVDV may associate with distinct mitophagy pathway.

Accumulating evidence indicates the role of mitophagy in regulating the inflammatory responses (37). Then, we examined whether BVDV-induced ferroptosis followed by mitophagy induction plays a role in inflammatory responses during BVDV infection. We detected the expression levels of proinﬂammatory genes such as interleukin-18 (IL-18), IL-1β, and interferon beta (IFN-β) in BVDV-infected cells in the presence or absence of Fer-1. Our data showed that CP BVDV infection signiﬁcantly stimulated the expression levels of IFN-β, IL-18, and IL-1β compared to mock-infected cells (Fig. 8C through E). Intriguingly, NCP BVDV infection has no or little effects on the expression levels of these genes compared to mock-infected cells (Fig. 8C through E). Fer-1 treatment significantly decreased the expression levels of proinﬂammatory genes in CP BVDV-infected cells while has no significant effects on NCP BVDV-infected cells (Fig. 8C through E). Furthermore, to determine the role of mitophagy in inflammatory responses in BVDV-infected cells, cells were transfected with siRNA targeting Parkin to specifically inhibit mitophagy (38, 39), followed by BVDV infection. Western blot analysis showed that Parkin protein expression was effectively inhibited by transfection of siRNA (Fig. 8F). Although knockdown of Parkin had no significant effects on the expression of IFN-β (Fig. 8F), IL-18 (Fig. 8G), and IL-1β (Fig. 8H) in CP BVDV-infected cells compared to control siRNA-transfected cells, an increased level of cytokines indicated was detected in NCP BVDV-infected cells pretransfected with siParkin compared to control siRNA-transfected cells (Fig. 8F through H). Thus, CP BVDV induced ferroptosis-mediated mitochondria damage and, subsequently, the induction of defective mitophagy, contributing to inflammatory cytokines expression. In contrast, ferroptosis-mediated mitochondria damage in NCP BVDV-infected cells was cleared via complete mitophagy, which suppressed inflammatory responses.

## DISCUSSION

Ferroptosis is frequently found to be induced under pathological conditions, including neurotoxicity, neurodegenerative diseases, acute renal failure, hepatic and heart ischemia/reperfusion injury, and cancer cell death (40, 41), while the role of ferroptosis in viral infection and pathogenesis is poorly understood. In this study, both CP and NCP BVDV infection decreased the MDBK cells viability. Concurrently, indicators of ferroptosis such as iron overload, ROS accumulation, and lipid peroxidation occurred in both biotypes of BVDV-infected cells. Moreover, inhibition of ferroptosis by Fer-1 or overexpressing GPX4 abrogated induced ferroptosis by BVDV, as evidenced by the findings that cell viability was increased along with decreases in lipid peroxidation. Furthermore, our findings suggest that both CP and NCP BVDV-induced ferroptosis enhanced viral replication. A possible explanation is that an increase in the intracellular ROS level can result in lipid peroxidation and cell death, which are beneﬁcial for virion release and systemic viral spread (42). Moreover, the increased ROS levels are tightly associated with virus-induced metabolic reprogramming, which has been found to support the replication of various viruses (18).

Ferroptosis results from the accumulation of cellular ROS. Importantly, it is the lipid ROS/peroxides, rather than cytosolic ROS, that unleash ferroptosis (8). However, where and how lipid ROS is generated during ferroptosis in response to virus infection have not been deﬁned. As the mitochondrion is a major organelle for cellular lipid ROS production, it appears that mitochondria should play a central role in ferroptosis (8). Here, we monitored lipid ROS accumulation in both biotypes of BVDV-infected cells. Indeed, upon viral infection, an increased level of ROS accumulation was observed not only in cytoplasm but also in mitochondria. Furthermore, when we examined subcellular localization of the mitochondrial lipid peroxidation probe by confocal imaging, we found that, in both biotypes of BVDV-infected cells and erastin-treated cells, the oxidized probe appeared in a distribution that signiﬁcantly colocalized with mitochondria rather than the cytoplasm. Thus, our findings provide compelling evidence that mitochondria are indeed a crucial player in ferroptosis induced by BVDV.

GPX4 is the most critical ferroptosis defense gene that encodes cytosolic, mitochondrial, and nucleolar isoforms, whose functions include scavenging free radicals and detoxifying various xenobiotics and, consequently, converting itself to its oxidized form, GSSG (16, 43). One of the key initial signals proposed to trigger ferroptosis is the inhibition of GPX4. In the present study, following both biotypes of BVDV infection, the expression and function of GPX4 in the mitochondria and intracellular organelles were greatly impaired, indicating that GPX4 may play a critical role in BVDV-induced ferroptosis. This result is consistent with the previous study where GPX4 was downregulated as well in virus-induced ferroptosis, including coronaviruses, human immunodeficiency virus, swine influenza virus, and Newcastle disease virus (NDV) (27, 44–46). Although the potential role of cytosolic GPX4 activity in various viruses-induced ferroptosis has been demonstrated, whether the mitochondrial GPX4 is an important component in ferroptosis during viral infection remains largely unknown. Here, more abundance of GPX4 in mitochondria was observed compared to that in cytoplasm either in CP or NCP BVDV-infected cells, and ferroptosis inhibitor Fer-1 treatment rescued the decreased expression of GPX4 in mitochondria, suggesting mitochondrial GPX4 may be a key point in ferroptosis caused by BVDV. The antioxidant transcription factor Nrf2 is a key negative player in ferroptosis (47). Almost all genes implicated in antiferroptosis thus far have been considered to be transcriptionally regulated by Nrf2, including GPX4, HO-1, and SLC7A11 (48). Our data showed that Nrf2 also was downregulated by either CP or NCP BVDV infection, implying that reduction of Nrf2 in response to BVDV infection may account for the rapid decrease of GPX4 and SLC7A11 expression in the process of ferroptosis. Mechanistically, the question how BVDV decreases Nrf2 expression remains an open issue.

Iron storage is equally important and may prevent cells from oxidative stress caused by an overload with redox-active free iron. In this context three iron storage proteins are known: FTH, FTL, and FtMt (49, 50). FTH as well as FtMt carries a ferroxidase activity, which allows the storage of ferric hydroxides (Fe^3+^) instead of reactive ferrous iron (Fe^2+^) (31, 50). Iron release from ferritins is regulated by a process known as ferritinophagy. Ferritinophagy is mediated by NCOA4, which directly binds FTH and transfers the complex to the autolysosome for degradation (50, 51). FtMt shows a high sequence homology to FTH, which implies a similar functionality (52). Recent studies showed that FtMt played inhibitory effects on oxidative stress-dependent ferroptosis via suppressing NCOA4-mediated ferritinophagy (53). Here, we show that a decreased FTH and FtMt expression in BVDV-infected cells is linked to endogenous NCOA4 expression. Knocking down NCOA4 by siRNA approach strongly enhanced the protein expression of FTH and FtMt. The observation that a decrease of endogenous NCOA4 either by genetic manipulation or by BVDV infection provokes a change of FtMt expression had not been reported before. Besides conﬁrming FTH as an established *NCOA4* target, we now provide evidence that FtMt is also under the control of NCOA4 during virus infection.

It has previously been demonstrated that ferroptosis is associated with dramatic morphological changes of mitochondria (8, 10). Moreover, excess iron and increased lipid peroxidation induced the mitochondrial damage and mitochondrial dysfunction (54, 55). Here, our data showed that both biotypes of BVDV induced ferroptosis, which was accompanied with mitochondria damage, especially in CP BVDV-infected cells, including a compromise of mitochondrial integrity and loss of MMP. Clearance of damaged and dysfunctional mitochondria via the induction of mitophagy is one of the self-limiting systems that can inhibit inflammation and mtDNA-cGAS-mediated type I IFN responses (56, 57). Here, we provide evidence that NCP BVDV induced complete mitophagy. In cells exposed to CP BVDV, however, accumulation of mitophagosomes did not lead to fusion with the lysosomes, likely due to a blockade in mitophagosome maturation and/or its clearance. Concurrently, increased expression of IL-1β, IL-18, and IFN-β compared to mock-infected cells was detected in CP BVDV-infected cells, while no significant different expression in these genes was detected between NCP BVDV- and mock-infected cells. These results are in agreement with previous *in vitro* studies demonstrating CP BVDV infection usually associated with inflammatory responses and NCP BVDV associated with no inflammatory responses (58–60). Importantly, inhibition of ferroptosis using Fer-1 abolished CP BVDV-induced inflammatory responses and interferon expression, while inhibition of mitophagy with siParkin transfection increased inflammatory cytokines and interferon expression in NCP BVDV-infected cells. These results may imply that both biotypes of BVDV induced ferroptosis-mediated mitochondria damage, and subsequently, mitophagy induction is tightly associated with inflammatory responses. Given that mitophagy acts as a brake on inflammasome signaling by removing dysfunctional mitochondria and that the attenuated inflammatory response may help viruses escape host antiviral immune defenses (61), it is reasonable to deduce that the distinct expression levels of inflammatory cytokines in response to CP or NCP BVDV infection may be closely associated with induction of different mitophagy pathways by biotypes of BVDV. Ever-increasing evidence demonstrates that some viruses have manipulated mitophagy to facilitate viral infection and pathogenesis (38, 62, 63). Although the molecular mechanism(s) underlying the different mitophagy pathways induced by biotypes of BVDV remain less understood, a functional cross-talk between biotypes of BVDV and induction of different mitophagy pathways is probably involved in mediating distinct pathogenesis of CP and NCP BVDV.

In summary, our results have established a novel link between mitochondria-mediated ferroptosis, mitophagy pathway, and distinct inflammatory responses in response to CP or NCP BVDV infection. To the best of our knowledge, this study is the first to show that mitochondria-mediated ferroptosis and mitophagy pathway are pathogenic mechanisms of biotype of BVDV-induced distinct pathogenesis, providing a novel therapeutic target for abrogating BVDV-mediated pathogenesis.

## MATERIALS AND METHODS

### Cell lines and viruses

HEK-293T cells were provided by China Center for Type Culture Collection (CCTCC, Beijing, China). Madin-Darby bovine kidney cells were provided by the American Type Culture Collection (ATCC CCL-22). The cells were cultured and maintained in Dulbecco’s modified Eagle’s medium (DMEM; Life Technologies Corporation, Gaithersburg, MD, USA) supplemented with 10% fetal bovine serum (Gibco, 10270–106), 100 IU/mL penicillin, and10 μg/mL streptomycin (Hyclone, SV30010) at 37°C in 5% CO_2_. The Chinese BVDV field strain HJ-1 (HJ-1, genotype 1b and CP type) was isolated from dead Holstein dairy cattle with mucosal disease. It was selected in this study because it produced a substantial CPE in MDBK cells and belonged to genotype 1b (GenBank accession no. JX065783 (64). The New York 1 strain of BVDV (genotype 1b and NCP type) obtained from China Veterinary Culture Collection Center was used in this study belonging to genotype 1b (GenBank accession no. FJ387232). Infected cells and supernatants were harvested and freeze thawed three times. Virus progeny production was determined by titration as described previously (65). The viral supernatants from MDBK cells were collected at the indicated time points after virus inoculation, and the TCID_50_ was calculated by the Reed-Muench method (66, 67).

### Antibodies and reagents

Primary antibodies used for western blot include: ARA70 Polyclonal Antibody (Immunoway Biotechnology, YT0302), Anti-Glutathione Peroxidase 4 antibody (Abcom, ab125066); Anti-Ferritin Heavy Chain antibody (Abclonal, A1144); Anti-Nrf2 antibody (Abclonal, A0674); Anti-Mitochondrial Ferritin antibody (Immunoway Biotechnology, YN4227); Anti-β-Actin Mouse Monoclonal Antibody (Invitrogen); Anti-p62 Polyclonal antibody (Proteintech Group, 18420-1-AP); anti-LC3B Antibody (Merck-Sigma-Aldrich, L7543); Anti-GAPDH antibody (Abcom, ab8245); Anti-VDAC1/Porin Polyclonal antibody (Proteintech Group, 55259-1-AP); Anti-MFN2 Polyclonal antibody (Proteintech Group, 12186-1-AP); Anti-HA Rabbit mAb (Cell Signaling Technology, 3724); Anti-Parkin antibody (Abcam,ab77924). Secondary antibodies: horseradish peroxidase (HRP)-conjugated goat anti-mouse IgG (Merck-Sigma-Aldrich, A9917), HRP-conjugated goat anti-rabbit IgG (Merck-Sigma-Aldrich, A0545).

Chemicals and reagents: MitoPeDPP (Dojindo, M466), Liperfluo (Dojindo, L248), FerroOrange (Dojindo, F374), DCFH-DA (Beyotime, S0033S), Mito-Tracker Red CMXRos (Beyotime, C1035), Mito-Tracker Green (Beyotime, C1048), JC-1 (Beyotime, C2003S), Ferrostatin-1 (Topscience, T6500), Erastin (Topscience, T1765), 3-methyladenine (MedChemExpress, HY-19312), carbonyl cyanide 3-chlorophenylhydrazone (CCCP, Merck-Sigma-Aldrich, C2759).

### Viral infection and cell treatment

MDBK cells were infected with CP BVDV (MOI = 5) and NCP BVDV (MOI = 10) or mock infected with phosphate-buffered saline (PBS) as control. Non-absorbed viruses were removed by repeatedly washing three times with PBS after 1 h incubation at 37°C in 5% CO_2_, then MDBK cells were maintained in DMEM supplemented with 2% FBS, 100 U of penicillin/mL, and 100 µg of streptomycin/mL at 37°C in 5% CO_2_. Cells were harvested for individual experiments at the indicated time points.

### Quantification of viral RNA

Real-time quantitative PCR assay was performed to detect virus copies in the same volume of medium. A series of dilutions of recombinant plasmid targeting the region corresponding to the N^pro^ gene of CP BVDV and NCP BVDV was utilized to generate a standard curve. Total RNA was extracted from BVDV-infected MDBK cells using Trizol reagent (Invitrogen, Carlsbad, CA, USA). An amount of 1 µg of total RNA was reverse transcribed to cDNA using Superscript III (Invitrogen). The qPCR reactions were performed in an ABI 7500 System (Applied Biosystems, Warrington, UK). Melt curves were performed to confirm the purity of the amplified products. The GAPDH gene was used to normalize Ct values. Each reaction was carried out in triplicate. Absolute quantities of BVDV RNA copies in different samples were calculated according to the standard curve.

### RNA interference

Small interfering RNAs targeting *NCOA4* (target site: CCAGCAAAGAAGACAGGAA), *Parkin* (target site: GCAGAGAAGTCGGGATCTACA), and *GPX4* (target site: GGAGUAAUGCAGAGAUCAA) were designed and synthesized by Tsingke, Inc. (Beijing, China). Small interfering RNAs were then used for silencing the target genes as described previously (66). Briefly, MDBK cells were transfected with 50 nM siRNA by using Tubrofect (Thermo Fisher, Waltham, MA, USA) according to manufacturer’s guidelines. After 24 h post transfection, MDBK cells were then infected with CP BVDV (MOI = 5) and NCP BVDV (MOI = 10), respectively. Cells were harvested for individual experiments at the indicated time post infection. The silencing efficiency was determined by immunoblotting analysis.

### Transmission electron microscopy

Semithin sections of cells were prepared and examined under a transmission electron microscope as described previously (68). MDBK cells were mock infected or infected with CP BVDV (MOI = 5) and NCP BVDV (MOI = 10) in 6 cm dishes. The cells were then washed three times with PBS and collected by centrifugation at 1,000 × *g* for 5 min, prefixed with 2.5% glutaraldehyde, then the cells were postfixed in 1% osmium tetroxide, dehydrated in series acetone, infiltrated in Epox 812 for a longer, and embedded. The semithin sections were stained with methylene blue, and ultrathin sections were cut with diamond knife, stained with uranyl acetate and lead citrate. Sections were examined with a Hitachi HT-7700 transmission electron microscope (Hitachi, Tokyo, Japan).

### Western blot analysis

Protein homogenates from the cells were extracted as previously described (66). Briefly, the cells were lysed for 20 min on ice-cold lysis buffer. The lysates were centrifuged at 12,000 × *g* for 20 min at 4°C to obtain a clear lysate. The protein content of each sample was determined using the BCA Protein Assay Kit (Thermo Scientific). Then, equal amounts of protein were separated on a 12% SDS-polyacrylamide gel and transferred to PVDF membranes (Merck-Millipore). Membranes were probed overnight at 4°C with primary antibodies. The bands were visualized using horseradish peroxidase-conjugated goat anti-mouse IgG (1:5,000, Merck-Sigma-Aldrich) or goat anti-rabbit IgG (1:5,000, Merck-Sigma-Aldrich) prior to the ECL protocol (Merck Millipore, Billerica, MA, USA). As an internal standard, all membranes stripped with primary antibodies were reprobed with anti-GAPDH and anti-ACTB antibody. Changes in protein expression were determined after normalizing the band intensity of each lane to that of GAPDH or ACTB. Signal was visualized using Konica SRX 101A developer (Konica Minolta Medical Imaging, Japan), and the Quantity One software (Bio-Rad, Mississauga, ON, Canada) was used for densitometrical analysis.

### Ferrous iron detection

The FerroOrange probe was used for flow cytometry and fluorescence imaging of intracellular Fe^2+^ (27). For the flow cytometry analysis, MDBK cells were infected with CP BVDV (MOI = 5) and NCP BVDV (MOI = 10) for the indicated time points. Meanwhile, the treatment group with erastin (10 µM) served as positive control. Then, cells were trypsinized and resuspended with FerroOrange working solution (1 µM) and incubated at 37°C for 30 min and then assessed using a BD FACS Aria III High Speed Cell Sorter (BD Biosciences, San Diego, CA, USA), followed by analysis with FlowJo software, version 10 (Tree Star, Ashland, OR, USA). For fluorescence imaging of intracellular Fe^2+^, FerroOrange (1 µM) dispersed in serum-free medium was added to the cells, followed by incubation for 30 min at 37°C. Then, the cells were treated with Mito-Tracker Green (2 µM) at 37°C for 30 min. The cells were then fixed with 4% paraformaldehyde for 45 min and washed again four times with PBS. Cells were photographed under a confocal microscope (A1R; Nikon, Japan). The fluorescence intensity was analyzed using Image J software.

### Mitochondrial membrane potential detection

MMP was measured using a JC-1 probe. The JC-1 probe was used for flow cytometry and fluorescence imaging of MMP. After various treatments of each group, MDBK cells infected with CP BVDV (MOI = 5) and NCP BVDV (MOI = 10) were harvested with trypsin, then suspended in PBS, and immediately stained with JC-1, according to the manufacturer’s instructions. Meanwhile, the treatment group with erastin (10 µM) served as positive control. Then, the samples were analyzed with a BD FACS Aria III High Speed Cell Sorter (BD Biosciences, San Diego, CA, USA), followed by analysis with FlowJo software, version 10 (Tree Star, Ashland, OR, USA). For fluorescence imaging of MMP, after various treatments of each group, MDBK cells infected with CP BVDV (MOI = 5) and NCP BVDV (MOI = 10) were immediately stained with JC-1 according to the manufacturer’s instructions. Meanwhile, the treatment group with erastin (10 µM) served as positive control. Cells were photographed under a confocal microscope (A1R; Nikon, Japan). The fluorescence intensity of each treatment group for both J-aggregates (red) and monomeric forms (green) of JC-1 was analyzed using ImageJ software (NIH).

### ROS measurement

The levels of intracellular ROS were determined by using a DCFH-DA probe for flow cytometry and fluorescence imaging. For the flow cytometry analysis, MDBK cells were infected with CP BVDV (MOI = 5) and NCP BVDV (MOI = 10) for the indicated time points. Meanwhile, the treatment group with erastin (10 µM) served as positive control. Then, cells were resuspended with DCFH-DA working solution (1 µM), then incubated at 37°C for 30 min, then assessed using a BD FACS Aria III High Speed Cell Sorter (BD Biosciences, San Diego, CA, USA), followed by analysis with FlowJo software, version 10 (Tree Star, Ashland, OR, USA). For fluorescence imaging of intracellular ROS, cells were seeded into a 12-well plate following BVDV infection. At the end of different treatments, cells were stained with DCFH-DA working solution (1 µM) for 30 min at 37°C, respectively. Then, the cells were treated with Mito-Tracker Red CMXRos (1 µM) at 37°C for 30 min. The cells were then fixed with 4% paraformaldehyde for 45 min and washed again four times with PBS. Cells were photographed under a confocal microscope (A1R; Nikon, Japan). The fluorescence intensity was analyzed using Image J software.

### Lipid peroxidation measurement

The levels of mitochondrial lipid peroxidation and intracellular lipid peroxidation were determined by using MitoPeDPP and Liperfluo probe for flow cytometry and fluorescence imaging. For the flow cytometry analysis, MDBK cells were infected with CP BVDV (MOI = 5) and NCP BVDV (MOI = 10) for the indicated time points. Meanwhile, the treatment group with erastin (10 µM) served as positive control. Then, cells were resuspended with MitoPeDPP working solution (1 µM) and Liperfluo working solution (5 µM), then incubated at 37°C for 30 min, then assessed using a BD FACS Aria III High Speed Cell Sorter (BD Biosciences, San Diego, CA, USA), followed by analysis with FlowJo software, version 10 (Tree Star, Ashland, OR, USA). For fluorescence imaging of mitochondrial lipid peroxidation, cells were seeded into a 12-well plate following BVDV infection. At the end of different treatments, cells were stained with MitoPeDPP working solution (1 µM) for 30 min at 37°C, respectively. Then, the cells were treated with Mito-Tracker Red CMXRos (1 µM) at 37°C for 30 min. The cells were then fixed with 4% paraformaldehyde for 45 min and washed again four times with PBS. Cells were photographed under a confocal microscope (A1R; Nikon, Japan). The fluorescence intensity was analyzed using Image J software.

### MDA assay

The detection of MDA concentration in cell lysates was performed strictly according to the manufacturer’s instructions (Beyotime, S0131S) (27). Malondialdehyde is the major product of lipid peroxidation (69). In this assay, lipid peroxidation is determined by the reaction of MDA with thiobarbituric acid to form a colorimetric product (532 nm), which is directly proportional to the MDA concentration. The absorbance at 532 nm was measured using a plate reader (Bio-Rad).

### GSH/GSSG assay

The detection of GSH/GSSG level in cell lysates was performed strictly according to the manufacturer’s instructions (Beyotime, S0053). Cells were infected with CP BVDV (MOI = 5) and NCP BVDV (MOI = 10) for the indicated time. The intracellular levels of reductive GSH were determined to detect reductive GSH concentrations in cell lysates. All steps in the procedure were based on the manufacturer’s instructions. The absorbance was measured at 405 or 415 nm. The experiment was performed as three independent replicates.

### Real-time quantitative PCR analysis

Real-time quantitative PCR assay was performed as previously described (70). Briefly, total RNA was extracted from MDBK cells using Trizol reagent (Invitrogen, Carlsbad, CA, USA). RNA was then reversed using Superscript III (Invitrogen). Real-time quantitative PCR was carried out using an ABI 7500 System (Applied Biosystems, Warrington, UK). The PCR cycling conditions were 30 s at 94°C followed by 40 cycles of 5 s at 94°C and 30 s at 60°C. Melt curves were performed to confirm the purity of the amplified products. Real-time quantitative PCR assay of bovine cytokine and GAPDH mRNAs was carried out and calculated using the 2^−ΔΔCT^method. Expression of the GAPDH gene was used to normalize cDNA levels for differences in total cDNA levels in the samples. Each reaction was carried out in triplicate. For the IFN-β, IL-18, IL-1β, and GAPDH primers used for real-time PCR, refer to previous studies (71, 72).

BVDV N^pro^ primers used in this study are as follows:

CP BVDV N^pro^, forward, 5′-CAACGCTAAAACTGCCACATAA -3′; CP BVDV N^pro^, reverse, 5′-CCCTGGTTTTAAATAGATTCCACTC-3′.

NCP BVDV N^pro^, forward, 5′- ATCCGCAGTCAACGCTAAAA -3′; NCP BVDV N^pro^, reverse, 5′- GGCCCTGGTTTTAAATAGATTCC -3′.

### Mitochondria isolation

The mitochondrial fractions were isolated using the Cell Mitochondria Isolation Kit (Beyotime, C3601) according to the manufacturer’s instructions. Briefly, the cells were harvested by washing twice in PBS, then digested and centrifuged at 200 × *g* for 10 min at room temperature. The cell pellets were resuspended with cold PBS and centrifuged at 600 × *g* for 5 min at 4°C. The treated cells were resuspended and incubated in 1–2 mL ice-cold mitochondrial lysis buffer (containing 1 mM PMSF) for 15 min. Subsequently, cell suspension was transferred to glass homogenizer and homogenized about 10–30 times. Then the cell homogenate was centrifuged at 600 × *g*, 4°C for 10 min. The supernatant was collected and carefully transferred to another tube and centrifuged at 11,000 × *g*, 4°C for 10 min to isolate the mitochondrial fraction (pellet) by removing the cytoplasmic fraction (supernatant). Then the mitochondrial fraction was subjected to perform western blot analysis.

### Analysis of mtDNA expression in cytosol

Mitochondrial separation was performed as previously described (39). Briefly, cells were harvested at individual time points post-infection, then washed with PBS, and DNA isolated using Quick-DNA Miniprep Kit (Zymo Research, D3025). mtDNA level was measured by comparing the relative levels of mtDNA with nuclear DNA by qPCR. The mitochondrial DNA amplicons were determined from two distinct segments of the mtDNA genes: mt-CO1 and mt-ND6. GAPDH was used as a nuclear amplicon as well as the internal control. Each reaction was carried out in triplicate. Primers used in this study are as follows: mt-CO1, forward, 5′-GTAGTTGTAACCGCACACGC-3′; mt-CO1, reverse, 5′-TTGCCTGCTAAGGGAGGGTA-3′.

mt-ND6, forward, 5′-AAAGCCGCAATCCCTATGGC-3′; mt-ND6, reverse, 5′- AGGGGCATTTGTTACTGGCT-3′.

### Lentivirus and stable cell line construction

Lentiviral production for overexpression was performed as follows. HEK-293T cells were seeded onto one plate of six-well plates. The following day, cells in each plate were transfected with 3 µg of pLenti-CMV-HA-Nrf2 or 3 µg of pLenti-CMV-GPX4, 2 µg of psPAX2 (gag, pol), and 1 µg of pMD2.G using 10 µL of Turbofect transfection reagent. Viral supernatants were collected 48 h after transfection and were filtered through 0.45-µm PVDF filters (Merck-Millipore). For lentiviral transduction, MDBK cells were seeded onto one plate of six-well plates and infected with appropriate viruses in six-well plates in the presence of 8 µg/mL polybrene (Merck-Sigma) at 37°C. After 24 h, medium containing puromycin (2 µg/mL) was added, and cells were selected for 72 h. The levels of overexpressed proteins were verified by western blot.

### Statistical analysis

The data are expressed as the means ± standard deviation of three independent experiments. The significance of the variability between the different treatment groups as calculated with one-way ANOVA, followed by Tukey’s multiple comparisons test using GraphPad Prism 6.0 software (GraphPad Software Inc., San Diego, CA, USA). *P < 0.05; **P < 0.01; ns, non-significant.
