# Correction for Li et al., “Mitochondria-mediated ferroptosis contributes to the inflammatory responses of bovine viral diarrhea virus (BVDV) *in vitro*”

**DOI:** 10.1128/jvi.00583-25

**Published:** 2025-05-13

**Authors:** Zhijun Li, Bao Zhao, Ying Zhang, Wenqi Fan, Qinghong Xue, Xiwen Chen, Jingyu Wang, Xuefeng Qi

## AUTHOR CORRECTION

Volume 98, no. 2, e01880-23, 2024, https://doi.org/10.1128/jvi.01880-23. Page 8, line 38: “(Fig. 6A and B)” should read “(Fig. 5A and B).”

Page 8, lines 46 and 48: “(Fig. 6C and D)” should read “(Fig. 5C and D).”

Page 8, line 53: “(Fig. 6E and F)” should read “(Fig. 5E and F).”

Page 10, line 4: “(Fig. 6H and I)” should read “(Fig. 5H and I).”

Page 10, line 5: “(Fig. 6J)” should read “(Fig. 5J).”

Page 10, line 14: “(Fig. 7A)” should read “(Fig. 6A),” “(Fig. 7B)” should read “(Fig. 6B),” and “(Fig. 7C)” should read “(Fig. 6C).”

Page 10, lines 22, 23, and 25: “(Fig. 5A)” should read “(Fig. 7A).”

Page 10, line 30: “(Fig. 5B)” should read “(Fig. 7B).”

Page 10, lines 31, 35, and 37: “(Fig. 5C)” should read “(Fig. 7C).”

Page 12, line 2: “(Fig. 5D)” should read “(Fig. 7D).”

Page 12, lines 5 and 8: “(Fig. 5E)” should read “(Fig. 7E).”

Page 15, lines 3 to 8: “Although knockdown of Parkin had no significant effects on the expression of IFN-β (Fig. 8F), IL-18 (Fig. 8G), and IL-1β (Fig. 8H) in CP BVDV-infected cells compared to control siRNA-transfected cells, an increased level of cytokines indicated was detected in NCP BVDV-infected cells pretransfected with siParkin compared to control siRNA-transfected cells (Fig. 8F through H).” should read “Although knockdown of Parkin significantly decreased the expression of IFN-β (Fig. 8G), IL-18 (Fig. 8H), and IL-1β (Fig. 8I) in CP BVDV-infected cells compared to control siRNA-transfected cells, an increased level of the indicated cytokines was detected in NCP BVDV-infected cells pretransfected with siParkin compared to control siRNA-transfected cells (Fig. 8G through I).”

We apologize for these errors. This correction does not affect the conclusions of the study.

